# *Agrobacterium rhizogenes*-mediated hairy root transformation as an efficient system for gene function analysis in *Litchi chinensis*

**DOI:** 10.1186/s13007-021-00802-w

**Published:** 2021-10-09

**Authors:** Yaqi Qin, Dan Wang, Jiaxin Fu, Zhike Zhang, Yonghua Qin, Guibing Hu, Jietang Zhao

**Affiliations:** State Key Laboratory for Conservation and Utilization of Subtropical Agro-Bioresources/Key Laboratory of Biology and Genetic Improvement of Horticultural Crops (South China), Ministry of Agriculture and Rural Affairs/Guangdong Litchi Engineering Research Center, College of Horticulture, South China Agricultural University, Guangzhou, China

**Keywords:** *Litchi chinensis*, *Agrobacterium rhizogenes*, Hairy root, Anthocyanin, LcMYB1

## Abstract

**Background:**

*Litchi chinensis* Sonn. is an economically important fruit tree in tropical and subtropical regions. However, litchi functional genomics is severely hindered due to its recalcitrance to regeneration and stable transformation. *Agrobacterium rhizogenes*-mediated hairy root transgenic system provide an alternative to study functional genomics in woody plants. However, the hairy root transgenic system has not been established in litchi.

**Results:**

In this study, we report a rapid and highly efficient *A*. *rhizogenes*-mediated co-transformation system in *L. chinensis* using Green Fluorescent Protein (*GFP*) gene as a marker. Both leaf discs and stem segments of *L. chinensis* cv. ‘Fenhongguiwei’ seedlings were able to induce transgenic hairy roots. The optimal procedure involved the use of stem segments as explants, infection by *A*. *rhizogenes* strain MSU440 at optical density (OD_600_) of 0.7 for 10 min and co-cultivation for 3 days, with a co-transformation efficiency of 9.33%. Furthermore, the hairy root transgenic system was successfully used to validate the function of the key anthocyanin regulatory gene *LcMYB1* in litchi. Over-expression of *LcMYB1* produced red hairy roots, which accumulated higher contents of anthocyanins, proanthocyanins, and flavonols. Additionally, the genes involving in the flavonoid pathway were strongly activated in the red hairy roots.

**Conclusion:**

We first established a rapid and efficient transformation system for the study of gene function in hairy roots of litchi using *A*. *rhizogenes* strain MSU440 by optimizing parameters. This hairy root transgenic system was effective for gene function analysis in litchi using the key anthocyanin regulator gene *LcMYB1* as an example.

## Background

Litchi (*Litchi chinensis* Sonn.) is a perennial fruit tree of the Sapindaceae family cultivated in tropical and subtropical zones of the world. It has been cultivated for more than 2300 years in China due to its delicious and nutritive fruits. In the past decade, substantial progress has been experienced into the molecular changes of flower and fruit development in litchi with the development of genetic and genomics tools [1–5]. However, gene functional characterization is severely hindered by the lack of an efficient regeneration and transformation protocol for litchi. Litchi is strong recalcitrance to in vitro regeneration. Different explants such as anthers, immature embryos, and leaves has been used to induce callus and somatic embryo, but only few of them regenerate successfully [6–8]. The main limitation of somatic embryogenesis is the large number of abnormal somatic embryos produced which cannot germinate or convert into normal plants [9].

*Agrobacterium tumefaciens*-mediated transformation has been widely used for plant genetic engineering and the study of gene function [10]. To date, successful *A*. *tumefaciens*-mediated transformation has been reported for several woody fruit crops, such as apple [11], peach [12], and citrus [13]. Puchooa [14] reported the *A*. *tumefaciens*-mediated litchi transformation protocols using leaf as explants, but no plantlets were regenerated from transformed calli. Padilla et al. [15] developed an *A*. *tumefaciens*-mediated transformation of 'Brewster' litchi with the *PISTILLATA* cDNA in antisense. In general, the transformation efficiency was low, and an efficient and reproducible transformation protocol is yet to be developed for litchi and other woody fruit crops [16].

*A. rhizogenes*-mediated hairy root transformation systems provide an alternative to species recalcitrant to transformation by *A. tumefaciens* [17]. Compared to *A. tumefaciens*-mediated transformation, *A. rhizogenes*-mediated transformation system is speedy, since transgenic hairy roots grow rapidly without a complex cultured process to regenerated plantlets [18, 19]. Therefore, *A*. *rhizogenes*-mediated transformation constitutes a simple, rapid and efficient method for the production of metabolites and the study of gene function in plants [20]. In grapevine, hairy roots were used to study the function of *VvMybPA1* or *VvMybPA2* on the regulation of proanthocyanidin biosynthesis [21]. Recently, Meng et al. [17] developed a simple, fast and efficient *A*. *rhizogenes*-mediated transformation for generating stable transgenic roots in living plants to facilitate functional studies in vivo. Subsequently, one-step generation of composite plants with transgenic roots by *A*. *rhizogenes*-mediated transformation has been established in peach, cucumber, and soybean [22–25]. In sweet potato, *A*. *rhizogenes*-mediated in vivo root transgenic system were shown to be a suitable system for the functional characterization of genes involved in salt tolerance [25]. However, the hairy root system has not been established in litchi. Therefore, an efficient hairy root transformation protocol is needed for rapid characterization of gene function in litchi.

In this study, we developed a reproducible, rapid and highly efficient *A*. *rhizogenes*-mediated hairy root co-transformation system for litchi. We optimized the transformation procedure using Green Fluorescent Protein (GFP) gene as a marker. Finally, we demonstrated the potential of the hairy root transgenic system to enable gene functional studies in litchi using the key transcriptional factor *LcMYB1* that regulates anthocyanin synthesis as an example.

## Materials and methods

### Plant materials and culture media

Seeds of *Litchi chinensis* Sonn. cv. ‘Fenhongguiwei’ were obtained from South China Agricultural University College, Guangzhou, China. Mature seeds were first washed in running water for 30 min. Thoroughly washed seeds were surface-sterilized with 75% ethanol for 1 min and dipped in sodium hypochlorite solution (1%) for 30 min, then rinsed 5 times with sterile water. Surface sterilized seeds were cultured on 1/2 MS medium [26] without sucrose. Culture was maintained at 25 ℃ in full light conditions (3000 lx) on a 16 h/8 h day/night cycle for seed germination.

### *Agrobacterium* strain and binary vectors

**Fig. 1:**
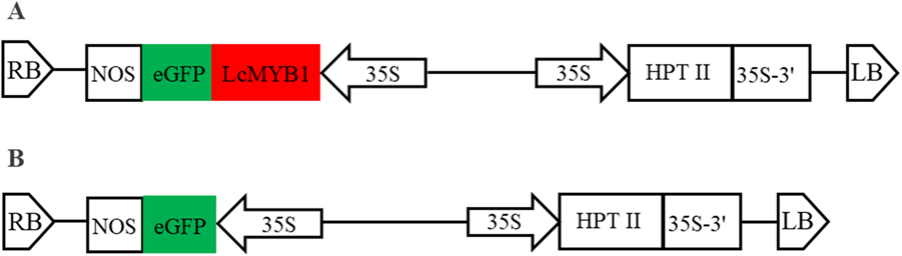
Schematic representation of the T-DNA region of the binary plasmid or pCAMBIA1300-LcMYB1-eGFP (**A**) and pCAMBIA1300-eGFP (**B**)

*Agrobacterium* *rhizogenes* strain MSU440 was used to induce transgenic hairy roots in litchi. The coding sequence of *LcMYB1* (accession number KY302802) without the termination codon was fused with the green fluorescent protein (eGFP) gene controlled by cauliflower mosaic virus (CaMV) 35S promoter in binary vector pCAMBIA1300 (Fig. 1). Binary vectors pCAMBIA1300-eGFP and pCAMBIA1300-LcMYB1-eGFP were introduced into *A*. *rhizogenes* strain by freeze–thaw method [27].

### *A*.* rhizogenes*-mediated transformation

*A.* *rhizogenes* strain MSU440 harboring binary vector pCAMBIA1300-eGFP or pCAMBIA1300-LcMYB1-eGFP was cultured in 600 μL YEP medium containing 50 mg L^−1^ kanamycin and 50 mg L^−1^ streptomycin and incubated overnight at 28 ℃ on a rotary shaker at 200 rpm. After the OD_600_ value reached to 0.6–0.8, bacterial cells were centrifuged at 5000 rpm for 8 min and re-suspended at different concentration (OD_600_ = 0.3, 0.5, 0.7, 0.9) in MS liquid medium containing 100 μM acetosyringone (AS). The resulting cell suspension culture was used in transformation.

Leaf discs and stem segments from 3- to 5- weeks-old litchi plants were submerged in bacterial solution for different incubation time (10, 20, 30, or 40 min) (infection concentration OD_600_ = 0.7), followed by removal of excessive liquid on sterile paper. Infected explants were then transferred onto filter papers wetted with the liquid MS medium with 100 μM AS and co-cultivated in the dark for 3, 5, and 7 days. After co-cultivation, infected explants were washed with sterile water and transferred onto MS medium containing 300 mg L^−1^ timentin and 300 mg L^−1^ carbenicilin.

### Fluorescence assay of regenerated hairy roots

The transgenic hairy roots were detected fluorescence using a stereomicroscope or scanning confocal microscopy (Zeiss, Germany) with filter sets for eGFP (525/50 nm). For histological assay of transgenic hairy roots, 0.5 cm long tip of hairy root was excised, and vertical sections were observed and imaged under a light microscope (DP27; Olympus, Japan).

### RNA isolation and PCR analysis

Hairy root RNA was extracted using RNAprep pure Plant Kit (TIANGEN, China) following the instruction. Then 1 μg total RNA was reverse-transcribed to cDNA using GoScript Reverse Transcription System (Promega, USA). RT-PCR was conducted using 2 × Taq Master Mix (Vazyme, China) following manufacturer’s instruction. Real-time quantitative PCR analysis (RT-qPCR) with SYBR Green Master Mix (Vazyme, China) was run in ABI 7500 Real-Time PCR System (Applied Biosystems, USA). The primers were listed in the Table 1. *LcActin* (accession number HQ615689) was used as internal control. Relative expression levels of candidate genes were calculated with the formula 2^−∆∆Ct^ [28]. All reactions were performed with three biological replicates.

**Table 1:** List of primer sequence

Gene	Forward primer (5′-3′)	Reverse primer(5′-3′)
For real-time qPCR		
*LcMYB1*	ACAGCAGAGACCATTTAGGG	TGATGTTTGTCCAAGCAGTTC
*LcPAL*	GCCAAGCAATTGATTTAAGG	CCACTTTGAGCAGATCCTTT
*LcC4H*	AGACGACTTGAACCACCGC	CCCGAACTCGACTCCCTGT
*LcCHS*	GACATTGTGGTGGTGGAGGT	TATTTAGCGAGACGGAGGAC
*LcCHI*	CGGAGTTTACTTGGAGGATGT	CAGTGACCTTCTCAGAGTATTG
*LcF3H*	GGTGGATAGATGTGACAAAGGAGT	GGTTGTGGGCATTTTGGATAGTA
*LcF3’H*	GCTCCGTCCATCTCTTCTCC	CCGTCTCCGAACACTCTCC
*LcDFR*	GGACCCTGAAAACGAAGTAA	CACTCCAGCAAGTCTCATCA
*LcANS*	AGGAAGTTGGTGGTCTGGAAG	CCGTTGCTGAGGATTTCAATGGTG
*LcUFGT*	GCCACCAGCGGTTCCTAATA	ATGCCTCTGCTACTGCTACAATCT
*LcGST*	GAGCATAAGCGTCCTGAGTTTC	TCCACGGTCCGCATACTTG
*LcLAR1*	TGAGAGTAGAGAAATCCGAATG	ATGACCTGTTTGGTAGAGAGAA
*LcLAR2*	ATGGCACCGTCAAAGCATAC	TTTCCCACAGAGAAGCAAGC
*LcANR*	AGGGCTATGTTGTTCACACTAC	AGCAAAATTGACTGGTGTTG
*LcFLS1*	GAGAGAGGTGGTGGACAAGT	TTGGAACAAGAACGGTGAG
*LcFLS2*	AGCCCATTGAAGGTGTAAAG	CTTGGAGCCGTTGGATTA
*LcACT*	ACCGTATGAGCAAGGAAATCACTG	TCGTCGTACTCACCCTTTGAAATC
For analysis of PCR		
*eGFP*	ATGGTGAGCAAGGGCGAGGAGCTGTTCACC	TTACTTGTACAGCTCGTCCATGCCGAGAGTGATCCC
*rol B*	GCTCTTGCAGTGCTAGATTT	GAAGGTGCAAGCTACCTCTC
*hpt II*	CTATTTCTTTGCCCTCGGACGAGTGCTGGGGCGT	ATGAAAAAGCCTGAACTCACCGCGACGTCTGTCGA
*Vir D*	ATGTCGCAAGGCAGTAAG	CAAGGAGTCTTTCAGCATG

### Analysis of anthocyanin, flavonols and proanthocyanidins content

For anthocyanin quantitation, 50 mg of hairy roots was extracted with a solution of mix methanol, water and concentrated hydrochloric acid (85:12:3) overnight at 4 ℃. Total anthocyanin content was measured using absorbance at 530 nm that have been diluted with pH 1.0 and 5.0 buffers.

For flavonols and proanthocyanidins extraction, hairy roots was extracted using methanol following the method described by Tiberti et al. [29] and Fiorella et al. [30], respectively. Flavonols and proanthocyanidins content were measured using absorbance at 510 nm and 500 nm, respectively.

### Statistical analysis

All experiment were conducted with three times. All the statistical analysis were performed using a t-test by SPSS software. Significance was indicated by asterisks * (*P* < 0.05).

## Results

### Transgenic hairy root induction from litchi

**Fig. 2:**
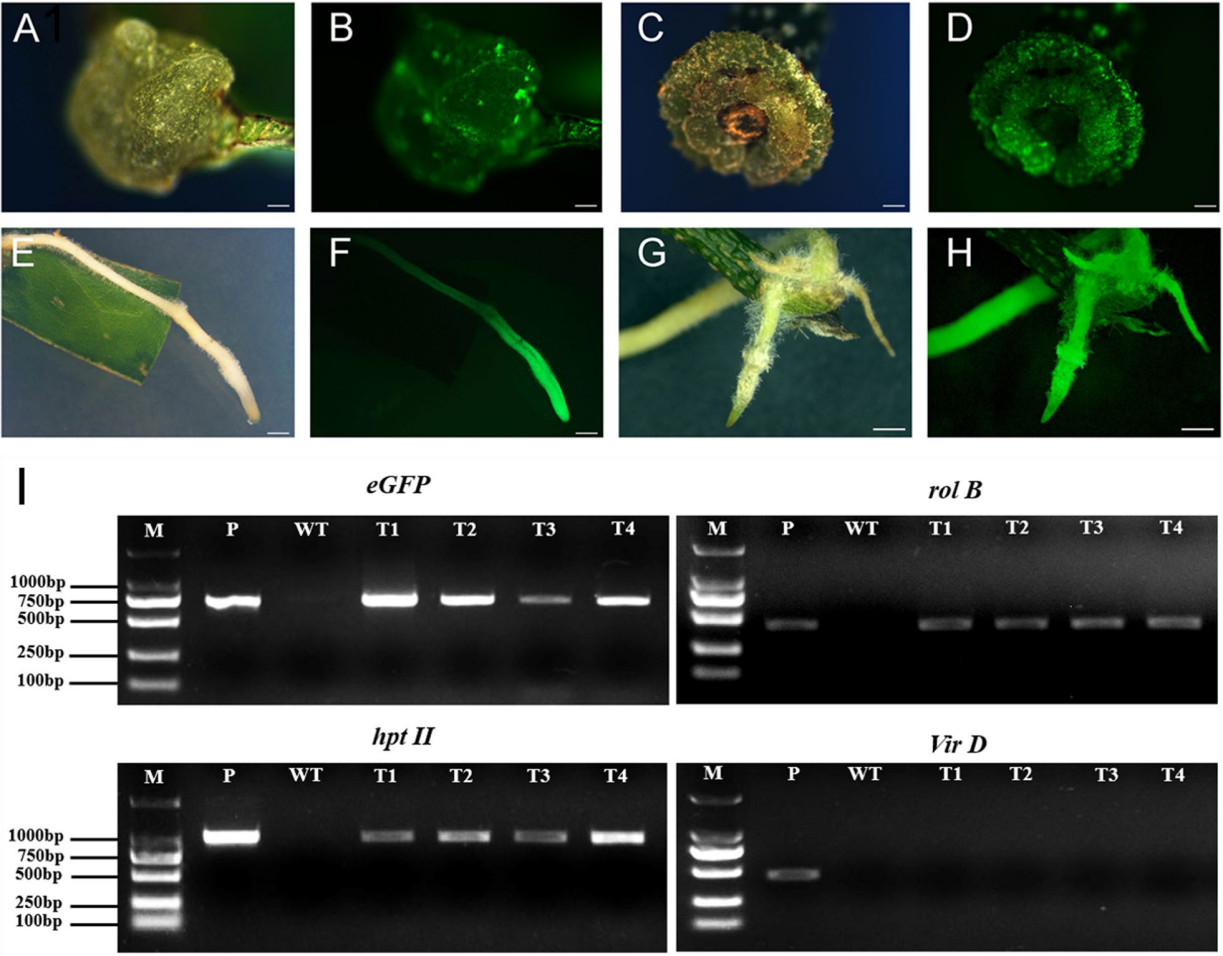
*A*. *rhizogenes* mediated transformation of litchi. Calli (**A**) and GFP fluorescence (**B**) induced from leaf discs. Calli (**C**) and GFP fluorescence (**D**) induced from stem fragments. Transgenic hairy root (**E**) and GFP fluorescence (**F**) from leaf explants. Transgenic hairy root (**G**) and GFP fluorescence (**H**) from stem explants. PCR analysis (**I**) for *eGFP*, *rol B*, *hpt II*, and *Vir D* in independent transgenic hairy roots

Leaves and stems from 4-week-old seedlings were used as transformation explants. Approximately 20 days after infection, calli appeared around the cutting site of leaves and stems (Fig. 2A, C). After about 5 weeks, small hairy roots appeared from the calli and elongated (Fig. 2E, G). The co-transgenic roots were detected by screening for GFP fluorescence signal. GFP expression was initially weak and only observed in a few cells of the calli from leaf and stem explants (Fig. 2B, D). Later, stronger GFP expression was observed in the entire transgenic hairy roots (Fig. 2F, H). To further verify that the co-transgenic nature of hairy roots regenerated, PCR analysis was carried out from 4 randomly selected independent transgenic hairy roots. Bands representing the *eGFP* and *hpt II* were detected in the transgenic hairy roots with GFP fluorescence, but not in the control (Fig. 2I). Similarly, the *rol B* gene responsible for directing hair roots differentiation was also detected in transgenic hairy roots. In addition, *Vir D* gene required for the T-DNA transfer and processing but located outside of T-DNA region of Ri plasmid was absent in transgenic hairy roots (Fig. 2I), showing that there was no *A*. *rhizogenes* contamination. In summary, a successful *A*. *rhizogenes*-mediated hairy roots co-transformation system was established in litchi.

### Optimization of *A*.* rhizogenes*-mediated litchi transformation

**Fig. 3:**
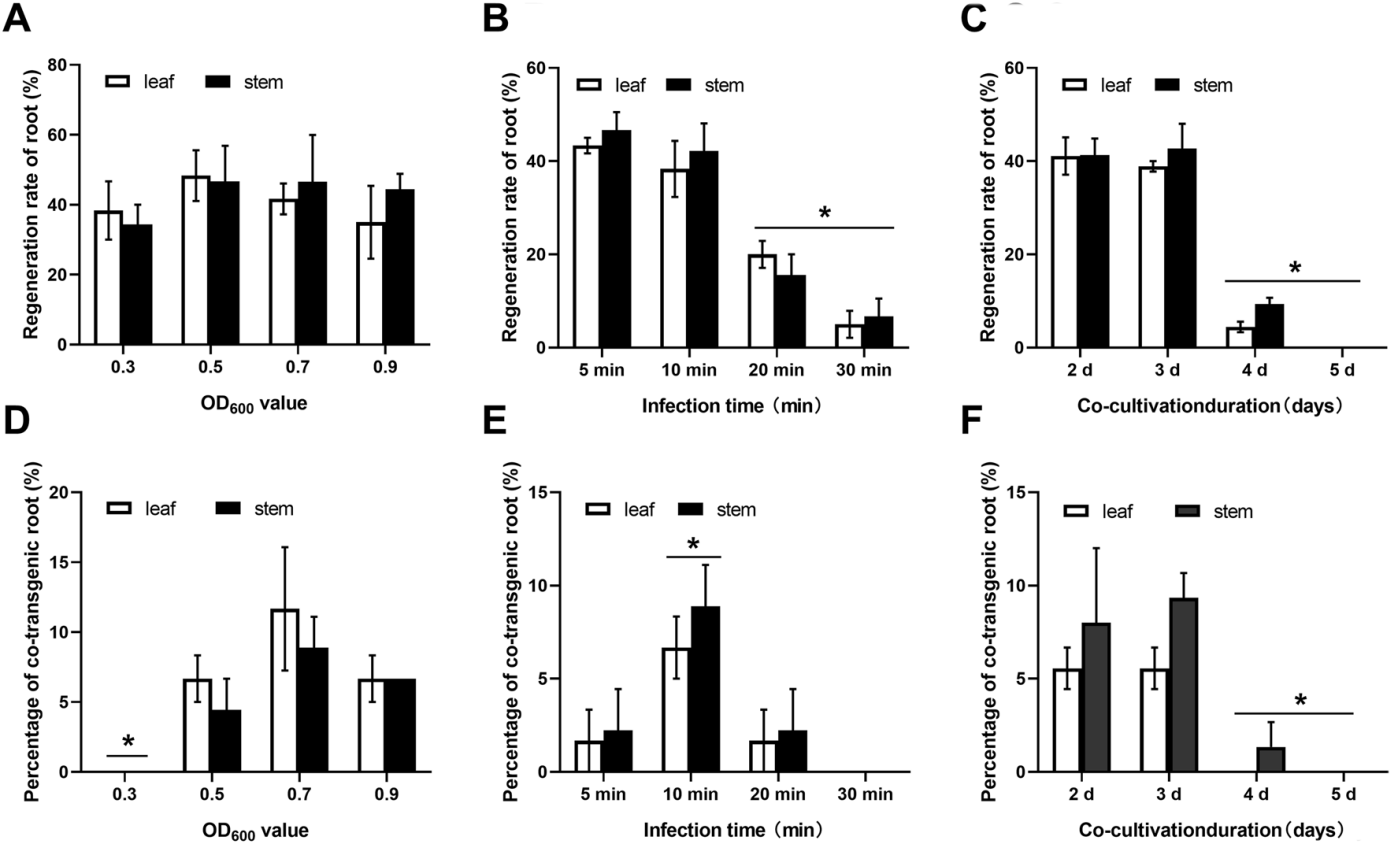
Optimization of *A*. *rhizogenes*-mediated litchi transformation using leaf and stem as explants. The regeneration rate of hairy roots (**A**–**C**) and co-transformation efficiency (**D**–**F**) after infecting different OD_600_ values, times, and co-cultivation duration of *Agrobacterium* strain MSU440, respectively. * indicate significant differences (*P* < 0.05)

In order to optimize the *A*. *rhizogenes* co-transformation efficiency, *A*. *rhizogenes* concentration, infection time, and duration of co-cultivation were tested. Firstly, various concentrations of *A*. *rhizogenes* were compared with the infection time of 10 min and co-cultivation for 3 days. When *A*. *rhizogenes* concentration of OD_600_ value ranged from 0.3 to 0.9, the hairy root regeneration rate of leaf and stem explants ranged from 35 to 48%, but there was no significant difference between leaf and stem explants (Fig. 3A). The highest co-transformation efficiency of leaf and stem were 8.33% and 8.89%, respectively (Fig. 3D). Subsequently, different infection times were tested with the *A*. *rhizogenes* concentration of OD_600_ = 0.7 and co-cultivation for 3 days. The hairy root regeneration rate of leaf and stem explants decreased significantly with the increasing infection times (Fig. 3B). Co-transformation efficiency increased significantly from 5 to 10 min, then decreased significantly. The co-transformation efficiency in 10 min of leaf and stem were 6.67% and 8.89%, respectively (Fig. 3E). Finally, various co-cultivation times were evaluated with the *A*. *rhizogenes* concentration of OD_600_ = 0.7 and infection time for 10 min. The results indicated that the hairy root regeneration rate and co-transformation efficiency of leaf and stem at the 2 days and 3 days co-cultivation duration was significantly higher than that of 4 days and 5 days (Fig. 3C, F). When co-cultivation duration for 3 days, the highest co-transformation efficiency of leaf and stem were 5.56% and 9.33%, respectively. Taken together, these results indicated the optimal infection condition was *A*. *rhizogenes* concentration of OD_600_ = 0.7, infection time for 10 min, and co-cultivation for 3 days.

### Gene functional analysis using the hairy root transgenic system

**Fig. 4:**
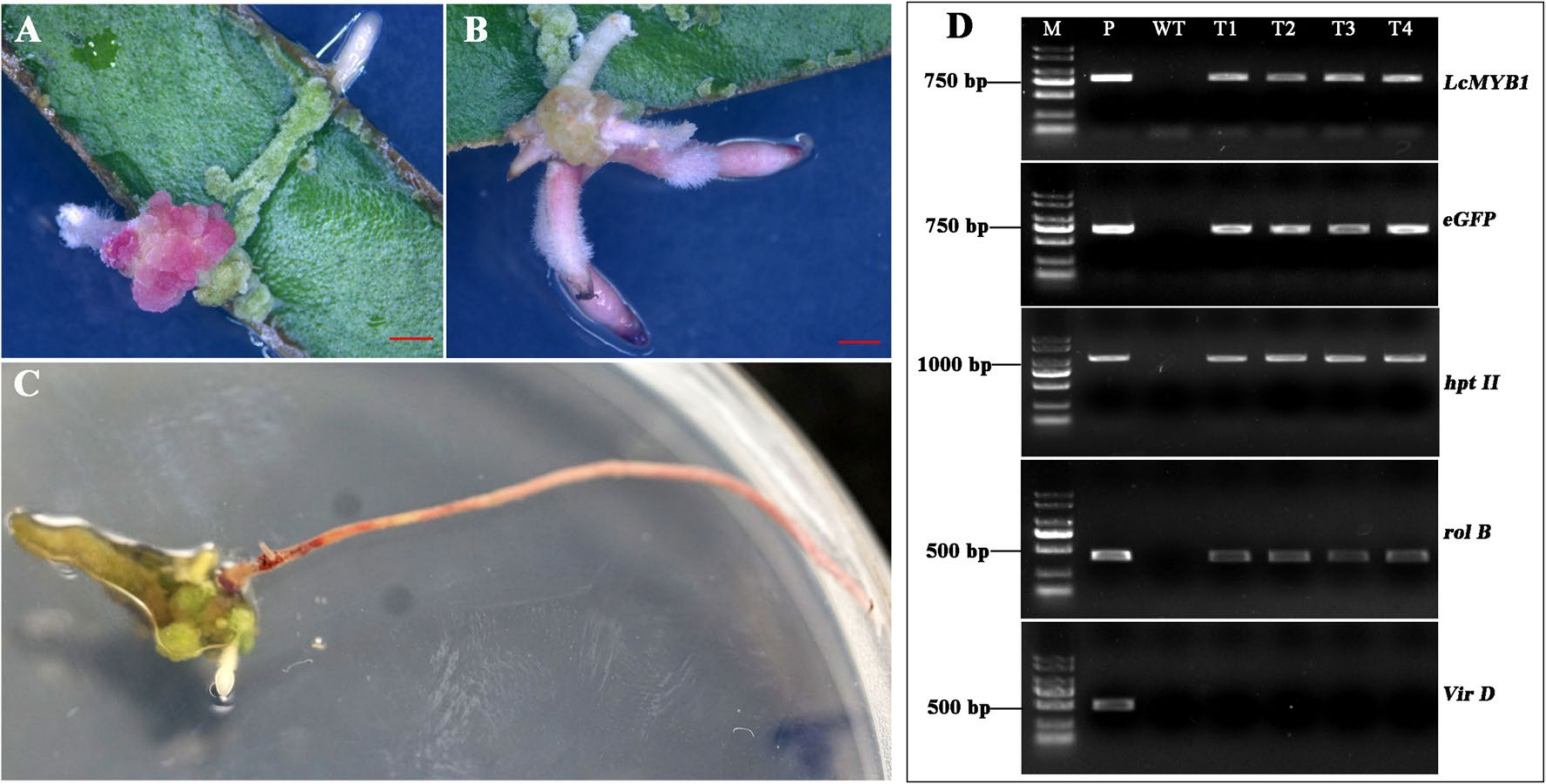
Phenotype and molecular validation of transgenic hairy roots overexpressing *LcMYB1* in leaf discs. Callus (**A**) and transgenic hairy roots (**B**, **C**) induced from leaf explants. PCR analysis (**D**) of *LcMYB1*, *eGFP*, *rol B*, *hpt II*, and *Vir D* in independent transgenic hairy roots for validation of overexpressing *LcMYB1*

To further confirm that the hairy root transgenic system is suitable for conducting gene functional analysis, *LcMYB1*, the key transcription factor that regulates anthocyanin biosynthesis in litchi, was co-transformed in the hairy root system. The coding sequence was cloned into pCAMBIA1300-eGFP vector to generate the C-terminal GFP fusion protein (Fig. 1A). Then the pCAMBIA1300-LcMYB1-eGFP vector was transferred into *A. rhizogenes* strain MSU440 and used for transformation. Red callus appeared after 3 weeks of overexpressing *LcMYB1* in leaf and stem using the hairy root system (Fig. 4A). After 1 more week, red hairy roots were regenerated from the red callus (Fig. 4B, C). PCR analysis revealed that *LcMYB1*, *eGFP*, *hpt II*, and *Vir D* genes were detected in the red hairy roots, but absent in the control (Fig. 4D).

**Fig. 5:**
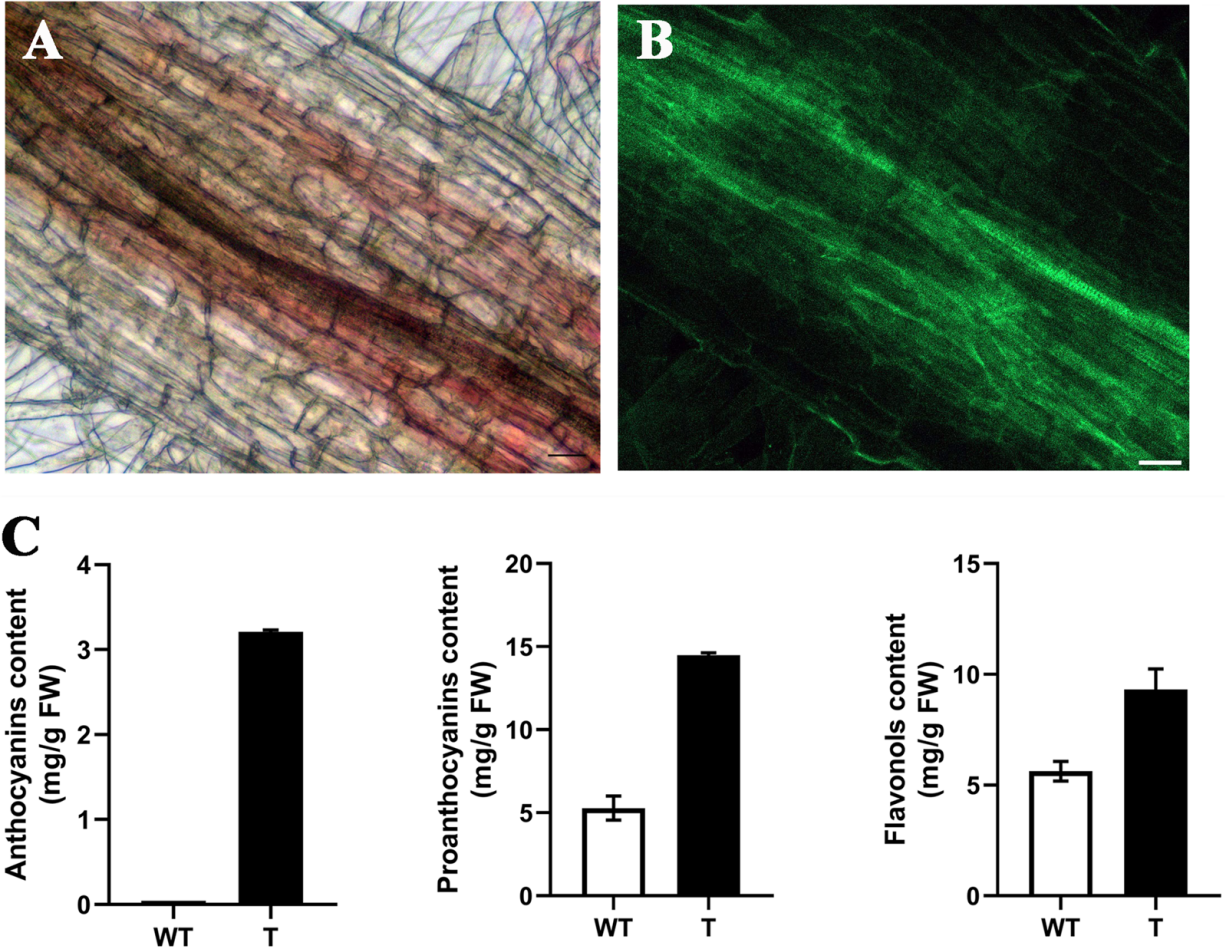
Color observation and quantitative analysis of anthocyanins, proanthocyanins, and flavonols contents in red hairy roots. Red coloration (**A**) was seen in the transgenic hairy roots exhibiting GFP expression (**B**). **C** Relative anthocyanin, proanthocyanins, and flavonols contents of wild-type (WT) and transgenic red hairy roots (T)

Upon closer inspection, the cells of red hairy roots were visualized in red by accumulating anthocyanin (Fig. 5A) and all the red cells exhibited stable GFP expression (Fig. 5B). In contrast, anthocyanins and GFP expression were absent in the non-transgenic roots. Quantitative analysis showed that contents of anthocyanins, proanthocyanins, and flavonols were significantly higher in red hairy roots. The relative anthocyanins, proanthocyanins, and flavonols contents in red hairy roots were, respectively, 69, 3, and 2 times greater than the control roots (Fig. 5C).

**Fig. 6:**
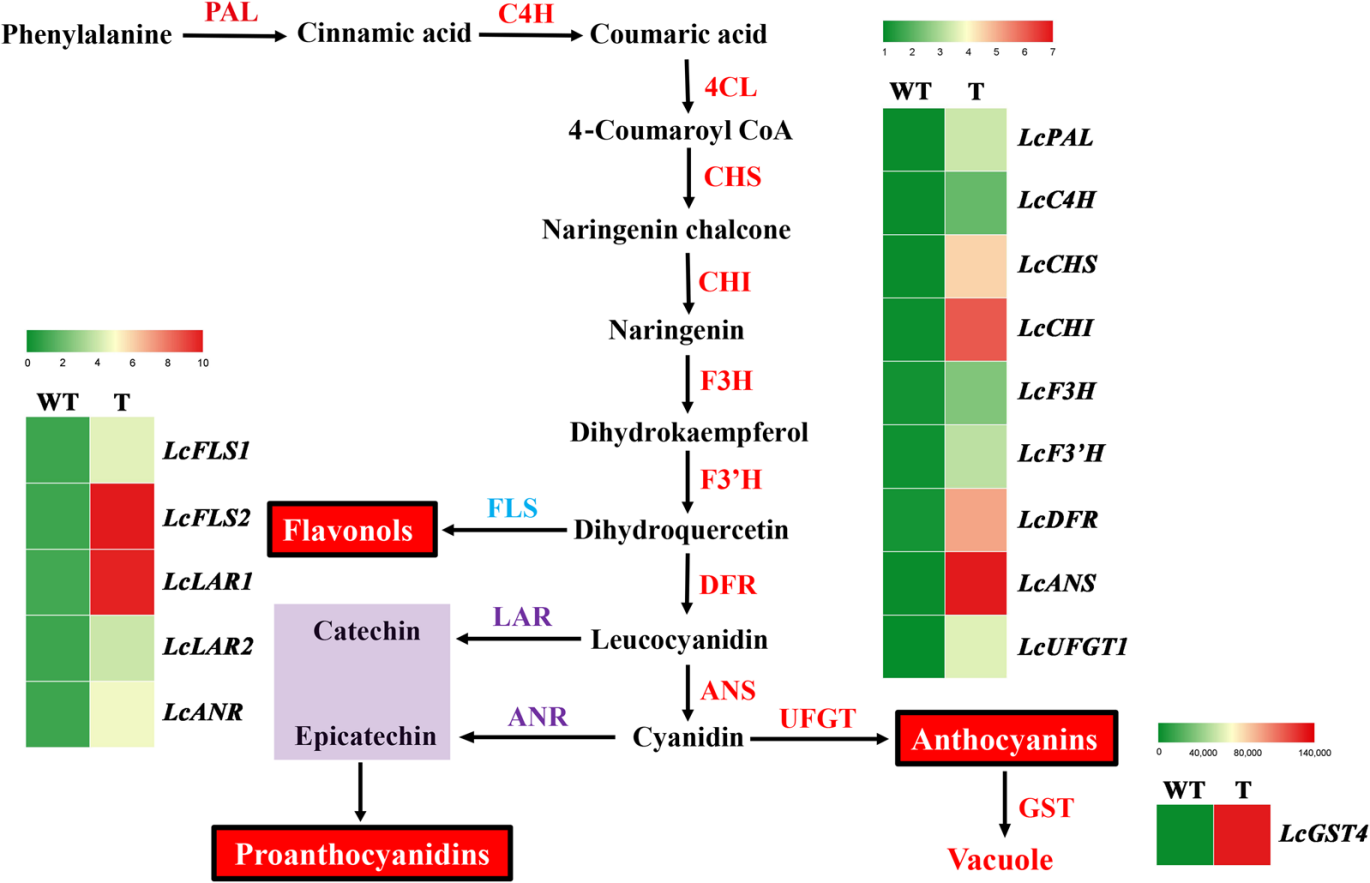
The expression levels of key enzyme genes involved in flavonoid pathway analysed using RT-qPCR were shown in hotspot map. *PAL* phenylalanine ammonia lyase, *C4H* cinnamate-4-hydroxylase, *4CL* 4-coumaroyl-coA synthase, *CHS* chalcone synthase, *CHI* chalcone-flavanone isomerase, *F3H* flavanone-3-hydroxylase, *F3′H* flavonoid-3′-hydroxylase, *FLS* flavonol synthase, *DFR* dihydroflavonol-4-reductase, *LAR* leucoanthocyanidin reductase, *ANR* anthocyanidin reductase, *UFGT* UDP-glucose:flavonoid-3-*O*glucosyltransferase, *GST* glutathione-S-transferase

To confirm the role of *LcMYB1*, the expression levels of its target genes in the flavonoid pathway, including *LcPAL* (LITCHI018600), *LcC4H* (LITCHI031057), *Lc4CL* (LITCHI002917), *LcCHS* (LITCHI020852), *LcCHI* (LITCHI027959), *LcF3H* (LITCHI006477), *LcF3'H* (LITCHI023381), *LcDFR* (LITCHI010261), *LcANS* (LITCHI022925), *LcUFGT* (LITCHI002457), *LcGST* (LITCHI008070), *LcFLS1* (LITCHI007338), *LcFLS2* (LITCHI011187), *LcLAR1* (LITCHI028570), *LcLAR2* (LITCHI005474), and *LcANR* (LITCHI029356) were detected using RT-qPCR. The results indicated that the expression of all these genes were up-regulated (Fig. 6). *LcGST4* (accession number KT946768), the gene involved in anthocyanin sequestration in litchi, was up-regulated more than 75281 folds. Additionally, the expression levels of *LcFLS1*/*2* involving in flavonols synthesis and *LcLAR1*/*2* and *LcANR* involving in proanthocyanidins synthesis were also up-regulated. Taken together, these results confirmed that the transgenic hairy roots system mediated by *A. rhizogenes* could be used to study anthocyanin metabolism in litchi and offer a simple way to verify gene function in woody plants.

## Discussion

In the last two decades, increasing fruit tree genomic resources like genome sequences were available paving the way to the genetic engineering and molecular breeding of fruit plants for crop improvement [31]. It will be possible to identify the genes controlling the important horticultural traits. In litchi, transcriptome analysis was used to identify the key genes involving in anthocyanin biosynthesis [2, 32]. However, their function analysis was usually validated in model plants such as Arabidopsis and tobacco [4, 33, 34]. The major reason for this is the lack of litchi transformation system [35]. An effective transgenic technology will be crucial for genes functional analysis. Thus, our newly developed transgenic hairy roots system mediated by *A. rhizogenes* will advance functional genomics research in litchi.

To date, *A. tumefaciens*-mediated transformation systems were widely used for functional genomics in plants [10]. Recently, significant advances have been made in optimizing the genetic transformation of fruit trees [36, 37]. In litchi, only one report was successful in developing transgenic plants using *A*. *tumefaciens*-mediated method [15]. In this report, only gene expression levels of the four transgenic lines were analyzed without any phenotype analysis, as it needs 7–8 years (typical juvenile period for litchi) to obtain mature plants [15]. Therefore, *A*. *tumefaciens*-mediated transformation is time-consuming, recalcitrant nature and not efficient enough to allow the high-throughput for functional genomic research [23]. Besides *A*. *tumefaciens*, *A. rhizogenes* is also utilized on functional studies of genes, especially those involved in secondary metabolism and responses to environmental stresses [20]. Here, *A. rhizogenes*-mediated transformation was established in litchi using leaf and stem as explants. Similar report also showed that hypocotyl, leaf and shoot were suitable for *A. rhizogenes*-mediated transformation in peach [22].

Factors including *Agrobacterium* concentration, infection time, and co-cultivation time affect T-DNA delivery and its integration into the plant genome [38, 39]. In the present study, the co-transformation efficiency for leaf and stem explants were higher after they were co-cultivated for 2 or 3 days. With a longer period of 4 or 5 d, *A. rhizogenes* may overgrow, leading to explant cell damage, consequently, to low transformation efficiency [18]. There was no significantly difference of co-transformation efficiency for leaf and stem explants in litchi. Liu et al. [18] reported that the *A. rhizogenes* transformation efficiency varied with the type of explant of *Arachis hypogaea* and was highest with petioles.

So far, transgenic hairy roots system mediated by *A. rhizogenes* has already been widely applied for many purposes including metabolism, root biology, and stress response [20, 25, 40]. Here, we used the established transgenic hairy roots system to study the gene function of *LcMYB1* which regulates anthocyanin biosynthesis in litchi. The result indicated that overexpression of *LcMYB1* could induce anthocyanin accumulation and produce red hairy roots. Previously, anthocyanins were accumulated in the tobacco hairy roots overexpressing *LcMYB1* [41]. RT-qPCR showed that *LcMYB1* could induce the expression of structural genes involved in anthocyanin biosynthetic in hairy roots of litchi. Therefore, transgenic hairy roots system was validated as an effective overexpression technique to study gene function in litchi. In addition, transgenic root system allows for silencing techniques or gene editing to be applied to plants that are recalcitrance to in vitro regeneration [17, 42]. These techniques in litchi need to be further explored.

## Conclusion

Here, we first established a rapid and efficient root transgenic system for litchi. The optimal parameters were infection by *A. rhizogenes* strain MSU440 at OD_600_ of 0.7 for 10 min and co-cultivation for 3 days. We validated this transformation system for study gene function in transgenic hairy roots by producing transgenic roots overexpressing *LcMYB1*, the key transcription factor that regulates anthocyanin biosynthesis in litchi. Transgenic roots demonstrated red color and increased flavonoid contents and displayed upregulation of flavonoid-related genes. These results will help to provide a simple and rapid method for gene function analysis in litchi.

## Data Availability

Not applicable.
