# Correction: Agrobacterium rhizogenes-mediated hairy root transformation as an efficient system for gene function analysis in *Litchi chinensis*

**DOI:** 10.1186/s13007-022-00973-0

**Published:** 2022-12-20

**Authors:** Yaqi Qin, Dan Wang, Jiaxin Fu, Zhike Zhang, Yonghua Qin, Guibing Hu, Jietang Zhao

**Affiliations:** State Key Laboratory for Conservation and Utilization of Subtropical Agro-Bioresources/Key Laboratory of Biology and Genetic Improvement of Horticultural Crops (South China), Ministry of Agriculture and Rural Affairs/Guangdong Litchi Engineering Research Center, College of Horticulture, South China Agricultural University, Guangzhou, China

**Correction: Plant Methods (2021) 17:103** https://doi.org/10.1186/s13007-021-00802-w

In the Funding information section, the Grant Number was incorrectly given as ‘No. 2019FYD1000900’ and should have read ‘No. 2019YFD1000900’. The original article [1] has been corrected.
